# Durable Tape-Cast Trilayer La_0.8_Sr_0.2_Ga_0.8_Mg_0.2_O_3‑δ_ Electrolyte with Infiltrated Electrodes for Intermediate Temperature Solid Oxide Fuel Cells

**DOI:** 10.1021/acs.jpcc.5c01421

**Published:** 2025-06-13

**Authors:** Daniel Sikstrom, Venkataraman Thangadurai

**Affiliations:** † Department of Chemistry, 2129 University of Calgary, Calgary, Alberta T2N 1N4, Canada; ‡ School of Chemistry, 7486 University of St Andrews, St Andrews, Fife KY16 9ST, U.K.

## Abstract

As global energy demands shift toward a sustainable alternative, hydrogen-powdered solid oxide fuel cells (SOFCs) offer a high-efficiency, low-emission solution for electrical energy conversion. However, performance limitations at intermediate temperatures (600–800 °C) necessitate advancements in electrolyte and electrode design. The present work presents the fabrication of a trilayer (porous/dense/porous) La_0.8_Sr_0.2_Ga_0.8_Mg_0.2_O_3‑δ_ electrolyte using a tape casting method, yielding a sintered structure with ∼55 μm thick, porous layers (∼55% porosity) and a ∼20 μm dense electrolyte supported by La_0.8_Sr_0.2_Ga_0.8_Mg_0.2_O_3‑δ_ rings. The porous La_0.8_Sr_0.2_Ga_0.8_Mg_0.2_O_3‑δ_ backbone is infiltrated with nominal chemical composition NdBaCoFeO_5+δ_ (NBCF) and a Ni–Gd-doped-Ce (Ni-GDC) anode. Electrochemical impedance spectroscopy, distribution functions of relaxation times, and equivalent circuit modeling identified an optimal NBCF loading of 1.58 mg/cm^2^, which minimizes charge transfer and diffusion resistance, reducing the area-specific resistance to 0.025 Ω cm^2^ at 800 °C. Full cell testing under SOFC conditions achieves a peak powder density of 400 mW/cm^2^ at 750 °C with low ohmic (0.11 Ω cm^2^) and polarization (0.33 Ω cm^2^) resistances.

## Graphical abstract

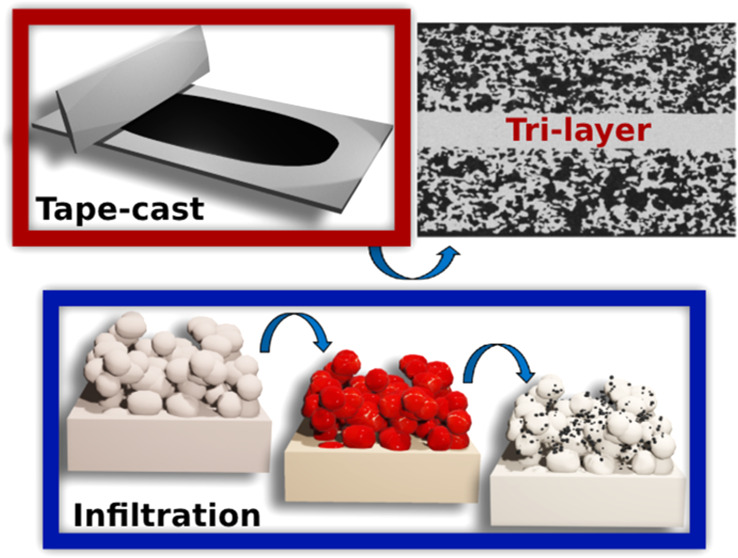

## Introduction

As more countries commit to decarbonizing, the demand for clean power generated from fossil fuel alternatives continues to increase.^1^ H_2_ plays a crucial role in decarbonizing the residential, commercial, and transportation sectors due to its high specific energy density and clean emissions, producing no CO_2_ during the utilization.^2–4^ However, challenges in H_2_ production and storage efficiency necessitates high power output when converting H_2_ into energy.^5^ Solid oxide fuel cells (SOFCs) convert H_2_ and O_2_ into electricity and water through a highly efficient chemical-to-electrical energy conversion process.^6,7^ The oxygen reduction reaction (ORR) occurs at the O_2(g)_-cathode-electrolyte interface, known as a triple-phase-boundary (TPB), where reduced O^2–^ ions are conducted through the de, reacting with hydrogen at the H_2(g)_-anode-electrolyte TPB.^3^ Both the ORR and H_2_ oxidation reaction (HOR) are multistep reactions involving gas diffusion, dissociated adsorption, and charge transfer. Traditional SOFCs operate at high temperatures (800–1000 °C) to overcome the sluggish kinetics of the ORR and ion conduction, which limit their long-term stability due to component degradation.^8^ Lowering the operating temperatures to the intermediate temperature (IT) range (typically 600–800 °C) reduces materials degradation but requires new electrode materials and designs to improve the kinetics and decrease internal resistance.

One approach to reducing ionic resistance involves minimizing the electrolyte thickness (5–10 μm) using thin films.^9^ Compared to other thin-film manufacturing techniques such as sputtering, screen-printing, and transfer-printing, tape casting is cost-effective, scalable, and compatible with a variety of structures and materials.^10,11^ The tape casting process involves dispersing ceramic electrolyte powder in a binder-plasticizer matrix, casting and drying the matrix to form a green tape, cutting and shaping the green tape, and sintering to create the electrolyte pellet.^12^ Improving the cathode kinetics for the ORR requires an extension of the TPB, which can be achieved in two ways. First, using mixed ionic-electronic conductors (MIECs) extends the TPB across the entire cathode surface creating a double-phase-boundary.^13–15^ Second, dispersing high-surface-area cathode nanoparticles onto the electrolyte introduces more ORR-active sites, increasing the TPB area.^16^ Solution infiltration offers a versatile, simple, and cost-effective method for producing nanoparticles at a lower temperature (<900 °C) compared to conventional solid-state synthesis (>1200 °C).^17^ This process dissolves metal precursors in solution, infiltrates the solution into a porous electrolyte backbone, evaporates the solution inside the backbone, and heats the infiltrated electrolyte to form nanoparticles.

Previous work shows that both single-phase Nd_0.75_Ba_0.25_Co_0.8_Fe_0.2_O_3‑δ_ perovskite and impure NdBaCoFeO_5+δ_ (NBCF) double-perovskite exhibit total electrical conductivity >300 S/cm at ITs when measured on dense pellets, and low area-specific resistance (ASR) (<0.15 Ω cm^2^ at 750 °C) and chemical compatibility with La_0.8_Sr_0.2_Ga_0.8_Mg_0.2_O_3‑δ_ (LSGM).^13,18,19^ This work fabricates thin, trilayer (porous/dense/porous) LSGM electrolytes using an organic tape casting method. A 4 mm wide, 0.5 mm thick LSGM rings surround the porous layer to enhance mechanical strength and prevent infiltrate crossover at the cell edge. Infiltrate solutions containing stoichiometric nitrate precursors for the NBCF double-perovskite cathode and Ni–Gd-doped-Ce (Ni-GDC) anode are infiltrated into the porous LSGM backbone and heated to produce nanoparticles. Electrochemical impedance spectroscopy (EIS), distribution of relaxation times, and equivalent circuit (EC) modeling between 600–800 °C analyze how the diffusion, dissociative adsorption, and charge transfer processes of the ORR for symmetric NBCF infiltrated cells vary with temperature and NBCF mass loading. In addition, current density–voltage (IV) measurement from 600–800 °C determine the peak power density (PPD) and benchmark the overall performance of the full cell, which consists of a trilayer LSGM electrolyte infiltrated with a NBCF cathode and Ni-GDC under SOFC conditions.

## Experimental Section

### La_0.8_Sr_0.2_Ga_0.8_Mg_0.2_O_3‑δ_ (LSGM) Green Tape Fabrication and Sintering

The dense layer tapes are prepared by adding 2.23 wt % of polyalkylene glycol, 0.76 wt % of benzyl butyl phthalate, 27.10 wt % of 2-butanol, and 4.92 wt % of poly­(vinyl butyral) to a 250 mL fluorinated ethylene propylene bottle and rolling for ≥24 h at 80 rpm until completely uniform. Then, 63.02 wt % of La_0.8_Sr_0.2_Ga_0.8_Mg_0.2_O_3‑δ_ (LSGM) (fuel cell materials), 1.97 wt % of α-terpineol, and before mixing, 5 mm Y-stabilized-Zr milling balls are added at 50 wt % of the total slurry weight. The slurry starts as a thick, biphasic mixture and must be rolled slowly at 30 rpm for 24 h to homogenize before increasing to 80 rpm for ∼6 days until complete deagglomeration occurs. The porous layer tapes are prepared using the same method, with 65% of the total ceramic content replaced by 19.26 wt % of graphite flakes (7–10 μm, 99%, ThermoFisher) alongside 29.35 wt % of LSGM. The tape-cast recipe changes are summarized in Table 1.

**Table 1:** Recipe for Porous and Dense La_0.8_Sr_0.2_Ga_0.8_Mg_0.2_O_3‑δ_ (LSGM) Green Tapes

	porous green tape		dense green tape	
	wt %	%v/v	wt %	%v/v
Poly(vinyl butyral)	4.51	5.62	4.92	8.64
Polyalkylene glycol	2.04	2.70	2.23	4.15
benzyl butyl phthalate	0.7	0.84	0.76	1.29
2-butanol	42.34	70.37	27.10	63.50
α-terpineol	1.81	2.62	1.97	4.03
LSGM	29.35	6.08	63.02	18.40
graphite	19.26	11.79	0.00	0.00

A small quantity of the corresponding slurry is poured across a Mylar sheet on a glass support and Doctor bladed (The Tape Casting Wearhouse) at a casting height of 0.1 mm for the thin, dense electrolyte layer, 0.5 mm for the porous layers, and 0.5 mm for the dense support rings. The wet tapes are left to dry in a dust-free environment for 24 h and removed from the Mylar sheeting using a sharp blade. After removal, the thin, dense green tape is cut into 19 mm diameter disks, the porous green tape is cut into 15 mm disks, and the dense support green tape is cut into 19 mm outer diameter and 15 mm inner diameter rings. The cut tapes are stacked according to (Figure 1); two dense electrolyte tapes are used to counteract small green tape defects from introducing pinholes in the final sintered pellet. Stacked green tapes, with graphite powder on the top and bottom, are placed between two flat crucibles and heated at a rate of 2 °C/min from room temperature to 500 °C and held for 2 h. From 500 to 1350 °C, the heating rate is then increased to 3 °C/min and held for 2 h. Finally, the sintered pellets are cooled to room temperature at 5 °C/min.

**Figure 1:**
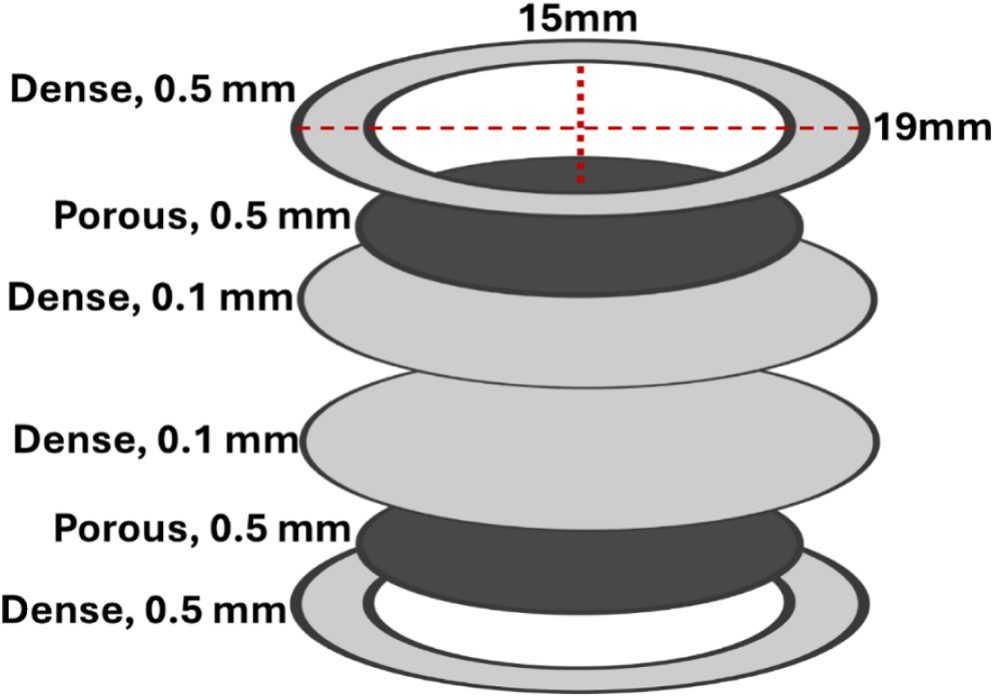
Schematic diagram of the stacked dense support ring, porous, and dense electrolyte green La_0.8_Sr_0.2_Ga_0.8_Mg_0.2_O_3‑δ_ (LSGM) tapes.

### NdBaCoFeO_5+δ_ (NBCF) Cathode and Ni–Gd-Doped-Ce (Ni-GDC) Anode Infiltration

The 0.1 M NdBaCoFeO_5+δ_ (NBCF) cathode infiltrate solution is prepared by adding 2.192 g of Nd­(NO_3_)·6H_2_O (99.9%, ThermoFisher), 1.31 g of Ba­(NO_3_)_2_ (99.999%, ThermoFisher), 1.46 g of Co­(NO_3_)_2_·6H_2_O (99%, ThermoFisher), and 2.02 g of Fe­(NO_3_)_3_·9H_2_O (99%, ThermoFisher) to a 50 mL volumetric flask and filled to volume with DI water. 3.24 g of Triton X-100 (Sigma-Aldrich) is added as a surfactant to prevent particle agglomeration, and the solution is homogeneous. The Ni/Gd-doped-Ce (Ni-GDC, 60/40 vol %) anode infiltrate solution is prepared according to previous work by adding 5.12 g of Ni­(NO_3_)_2_·6H_2_O (99%, ThermoFisher), 5.00 g of Ce­(NO_3_)_3_·6H_2_O (99.5%, Sigma-Aldrich), and 1.30 g of Gd­(NO_3_)_3_·6H_2_O (99.9%, ThermoFisher) to a 20 mL volumetric flask and filled to volume with DI water. 1.29 g of Triton X-100 is added as a surfactant to prevent particle agglomeration, and the solution is mixed until all nitrates are dissolved and the solution is homogeneous. The sintered LSGM pellets are infiltrated with 3 μL of the NBCF solution per cycle, repeated 4 times to fully saturate the porous backbone. The pellets are then placed under vacuum at a gauge pressure of 68 kPa to enhance the capillary action and accelerate solvent evaporation. For symmetric cells, the process is repeated on the opposite side of the LSGM pellet with the NBCF solution, while for full cells, the Ni-GDC solution is used instead. The infiltrated pellets are heated at 5 °C/min up to 800 °C, held for 1 h, and then cooled at 5 °C/min to room temperature. This process is repeated multiple times to increase the particle loading, with each infiltration depositing ∼0.53 mg/cm^2^.

### Thermogravimetric Analysis (TGA) Measurements

Thermogravimetric analysis (TGA) is performed using a Mettler Toledo TGA/DSC 1 Stare System from 25 to 1000 °C at a heating rate of 2.5 °C/min under an airflow of 40 SCCM.

### Scanning Electron Microscopy (SEM) Imaging

Prior to imaging, sintered LSGM pellets are mounted in epoxy, cut, and polished using 1000, 1500, 2000, 2500, 3000, and 4000 grit polishing paper followed by 15, 9, 6, 3, 1, and 0.05 μm monocrystalline diamond polishing suspension. Imaging is performed using a Quanta FEG250 scanning electron microscope equipped with a secondary backscattered electron detector at a voltage of 10 kV, a working distance of 10.9 mm, 750 × magnification, and horizontal field width of 199 μm and vacuumed to an absolute pressure of 7.03 × 10^–4^ Pa.

### High-Temperature Powder X-ray Diffraction (HT-PXRD) Measurements

High-temperature powder X-ray diffraction measurements (HT-PXRD) are obtained using a Bruker D8 Advance with a scintillation detector, variable temperature sample stage, Cu Kα radiation (40 kV, 40 mA, Kα_1_ = 1.54060 Å, Kα_2_ = 1.54439), 0.02° step size, and 0.2°/min scan speed over the 2θ range of 20–80°. The NBCF powder for HT-PXRD measurements was prepared by heating 15 mL of the NBCF infiltrate solution at 5 °C/min up to 800 °C, held for 1 h, and then cooled at 5 °C/min to room temperature.

### Electrochemical Impedance Spectroscopy (EIS) Measurements

A PARSTAT 4000A potentiostat/galvanostat is used for electrochemical impedance spectroscopy (EIS) measurements using alternating current (AC) with an amplitude of 10 mV over the frequency range 10^6^ – 10^–2^ Hz in ambient air under open-circuit voltage. Each data point was scanned 6 consecutive times and averaged with 10 points per decade and a 30 s measurement delay. Impedance spectra are area corrected using the external surface area of 0.95 cm^2^ for the porous LSGM backbone as there is no feasible way to accurately determine the surface area of all NBCF and NiO nanoparticles.

### Distribution of Relaxation Times (DRT) and Equivalent Circuit (EC) Modeling

A relaxation time is the time scale for a process in a perturbed system to reach equilibrium, as defined by the formula

1 $$\tau =\frac{1}{\omega_{c}}$$

where τ is the relaxation time and ω_c_ is the characteristic angular frequency of the process occurring at τ. In SOFC cathodes, which catalyze the oxygen reduction reaction (ORR), relaxation times are associated. Thus, analyzing the relaxation times of an electrochemical impedance spectra provides a deeper insight into which relaxation time and associated process contribute most significantly to the impedance response and which process is rate-limiting. However, the poor resolution of EIS often results in multiple relaxation times contributing to the impedance response at any frequency.^20^

A distribution function of relaxation times (DRT) can be employed to separate these overlapping contributions using the formula

2 $$Z{\left(\omega \right)}_{EXP}\approx Z{\left(\omega \right)}_{DRT}=R_{s}+R_{p}\int_{0}^{\infty }\frac{\Gamma \left(\tau \right)}{1+i\omega \tau }d\tau ,\mathrm{with}\;\int_{0}^{\infty }\Gamma \left(\tau \right)d\tau =1$$

where *Z*(ω)_DRT_ is the impedance generated from an infinite resistor-capacitor (RC) circuit and validated against the experimental impedance (*Z*(ω)_EXP_). *R*_o_ represents the frequency-independent ohmic resistance from the experimental impedance, *R*_p_ is the polarization resistance from the experimental impedance, Γ­(τ) is the distribution function of relaxation times, which describes the fractional contribution of a process at a given τ, ω is the externally applied angular frequency, and the 1 + iωτ models the real and ω-dependent imaginary component of the impedance response.^20,21^

Solving for Γ­(τ) requires the deconvolution of impedance data, which is assumed to be noninductive, and a technique such as evolutionary programming, the principle of maximum entropy, Fourier analysis, the Monte Carlo method, or regularized regression, which is used in this work due to it’s low sensitivity to noise, computational simplicity, and reproducibility of outputs.^20,22–25^ DRT tools is the chosen software for computing the DRT from the experimental EIS data using a Gaussian radial basis function for discretization, a first derivative Γ­(τ) penalty term to stabilize DRT oscillations, a regularization parameter of 8.13 × 10^–3^ (as recommended by Wan et al.), and the software default full-width-half-maximum coefficient of 0.5.^25,26^ The generated DRT is deconvoluted into 3 Gaussian distribution functions to separate the *R*_p_ into the low τ/HF (*R*_1_), mid τ/MF (*R*_2_), and high τ/LF (*R*_3_) contributions according to the formula

3 $$R_{i}=R_{p}\int_{0}^{\infty }g_{i}\left(\tau \right)d\tau$$

4 $$\sum_{i=1}^{3}\int_{0}^{\infty }g_{i}\left(\tau \right)d\tau =1$$

where *g_i_* (τ) is a deconvoluted Gaussian distribution function and *R_i_* is the resistance contribution of process *i*. The capacitance of process *i* (CPET*_i_*) is calculated from the formula

5 $${\mathrm{CPET}}_{i}=\frac{\tau_{i}}{R_{i}}$$

where τ*_i_* is the time constant of process *i*. An equivalent circuit (EC) model is generated from *R_i_* and CPET*_i_* calculated from the DRT and least-squares fitting is used to solve for the constant phase element phase factor (CPEP*_i_*). This factor, ranging between 0 and 1, quantifies the deviation from ideal capacitance behavior, where CPEP = 1 corresponds to an ideal capacitor. An HF inductor (L) was added to the EC to account for inductance effects in the HF -Z″ region and solved for using least-squares fitting. Finally, sequential least-squares fitting is used to further optimize each component of the EC while avoiding local minima trapping through the refinement of many simultaneous variables.

### Full Cell Assembly and Testing

Two Au wires are attached to the center of the cathode and anode using a small droplet of Ag ink (fuel cell materials), which is heated at 80 °C. The wired full cells are sealed onto ceramic tubing using Cermabond 552 (Aremco) and left to dry for 4 h at room temperature. The assembly is placed into a vertical furnace and heated at a rate of 2 °C/min to 93 °C, where it is held for 2 h. It is then further heated at a rate of 2 °C/min to 260 °C and held for another 2 h. Finally, the assembly is heated at 2 °C/min to 600 °C. Before testing, H_2_ is flows into the ceramic tubing at 100 SCCM to reduce NiO into Ni and expel the air. For full cell testing, 200 SCCM of H_2_ is flowed on the anode side, with ambient air being at the cathode side. A PARSTAT 4000A potentiostat/galvanostat is used for full cell EIS measurements using AC with an amplitude of 80 mV over the frequency range 10^6^–10^–1^ Hz under open-circuit voltage. Each data point was scanned 6 consecutive times and averaged with 10 points per decade and a 30 s measurement delay. A PARSTAT 4000A potentiostat/galvanostat is used for current density–voltage (IV) measurements scanned from open-circuit potential (OCV) to 0.4 V with a step height of 30 mV and a scan rate of 5 mV/s.

## Results and Discussion

### Binder and Plasticizer Ratio

It is found that approximately 75% of the total plasticizer should be Type 2. Below this percentage, cracks form in the tape during drying and the dried tape adheres to the Mylar support. Above this percentage, grains appear in the dried tape (Figure 2). Green tapes break during sintering at binder/plasticizer ratios of approximately 2 due to the high viscosity of the slurry, which necessitates reducing the solid loading to improve flowability. In contrast, green tapes with binder/plasticizer ratios of approximately 1.2 are overly brittle when dry and disintegrate after cutting. A binder/plasticizer ratio of 1.6 yields strong, defect-free green tapes that successfully pelletize during sintering. At this ratio, an optimal ceramic loading of ∼18%v/v is achieved; increasing the ceramic loading beyond this level leads to particle agglomeration, resulting in particles larger than the blade height, which can rip the tape during casting. Conversely, reducing the ceramic loading compromises the densification of the sintered pellets. The optimized tap-cast La_0.8_Sr_0.2_Ga_0.8_Mg_0.2_O_3‑δ_ (LSGM) recipe is provided in Table 1.

**Figure 2:**
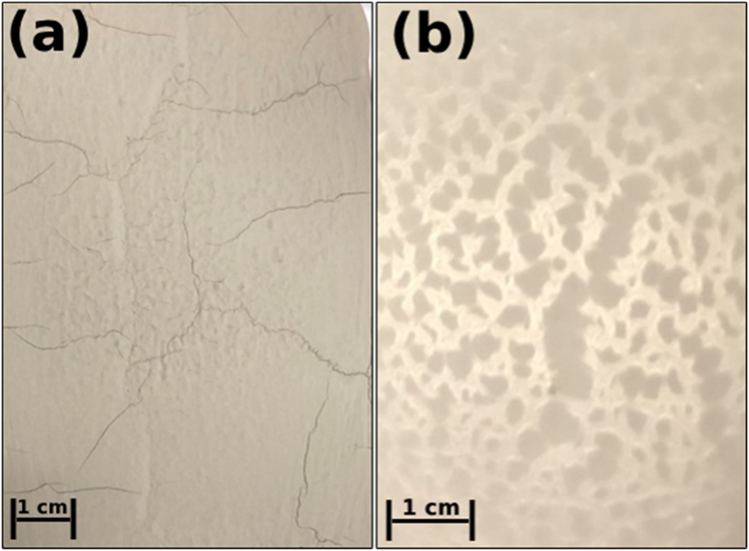
Surface of La_0.8_Sr_0.2_Ga_0.8_Mg_0.2_O_3‑δ_ (LSGM) green tapes from a slurry with (a) 60% of the total plasticizer being type 2 and (b) 90% of the total plasticizer being type 2.

### Green Tape Heating and Sintering

Thermogravimetric analysis (TGA) is used to determine the decomposition temperature of the porous green tape and the precursors to optimize the heating profile (Figure 3). Without the addition of the poly­(vinyl butyral) binder, benzyl butyl phthalate and polyalkylene glycol start volatilizing at 130 and 150 °C, respectively, and are completely in the gas phase at 280 and 290 °C. When combined with the poly­(vinyl butyral) binder, benzyl butyl phthalate begins to volatilize at 170 °C and volatilizes rapidly up to 315 °C, after which the rate slows and continues steadily until 425 °C. Beyond this temperature, the volatilization rate decreases significantly until 470 °C, where it resumes. Similarly, when polyalkylene glycol is combined with the poly­(vinyl butyral) binder, it begins to volatilize at 150 °C (the same as pure polyalkylene glycol) and steadily volatilizes until 325 °C. At this point, rapid volatilization occurs until 425 °C. Beyond this temperature, the volatilization rate decreases significantly until 460 °C, where it resumes. Both the poly­(vinyl butyral)- benzyl butyl phthalate and poly­(vinyl butyral)-polyalkylene glycol systems are fully converted to the gas phase by 525 °C. This analysis reveals that the final stages of debinding occur after the volatilization of the plasticizer/binder system in poly­(vinyl butyral)- benzyl butyl phthalate/polyalkylene glycol systems, enabling the ceramic particles to bind more closely without the particle separation from the plasticizers during the debinding process. The porous green tape begins to steadily volatilize at 150 °C until 230 °C with a 2 wt % loss and then rapidly volatilizes until 325 °C with a 7 wt % loss. This is equated to volatilization of the plasticizers and the plasticizer/binder system. The volatilization rate decreases significantly with steady volatilization and a 1 wt % loss until 340 °C, whereas rapid volatilization resumes until 395 °C with a 3 wt % loss. This is equated to the volatilization of the excess binder, which is not incorporated in the plasticizer/binder system. Finally, no volatilization occurs in the green tape between 395 and 825 °C where beyond this temperature, graphite combusts.

**Figure 3:**
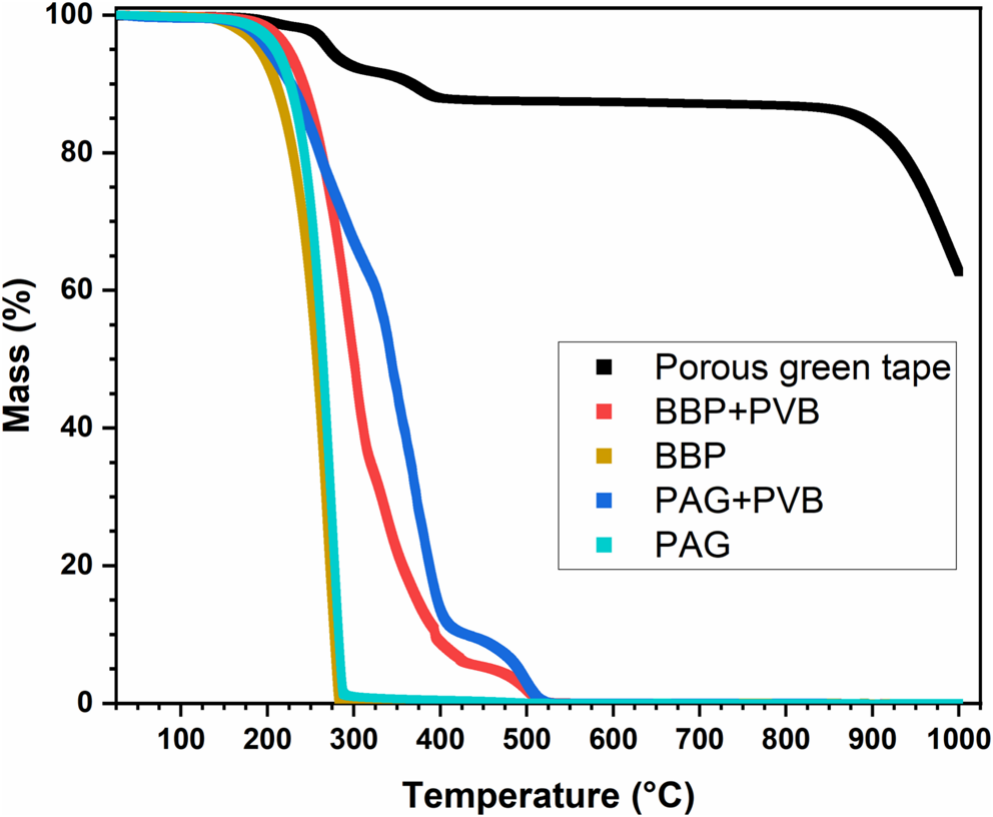
Thermogravimetric analysis (2.5 °C/min) in the air of a porous La_0.8_Sr_0.2_Ga_0.8_Mg_0.2_O_3‑δ_ (LSGM) green tape prepared according to Table 1, and other components used for tapes, including 20 wt % poly­(vinyl butyral) (PVB) in benzyl butyl phthalate (BBP), benzyl butyl phthalate (BBP), 20 wt % poly­(vinyl butyral) (PVB) in polyalkylene glycol (PAG), and polyalkylene glycol (PAG).

One-step, single-atmosphere sintering is achieved to form a porous/dense/porous, trilayer structure. Green tapes are heated at a rate of 2 °C/min from room temperature to 500 °C to ensure uniform shrinking during plasticizer/binder decomposition, and the temperature is held at 500 °C for 2 h to allow complete decomposition. From 500 to 1350 °C, the heating rate is increased to 3 °C/min. At a heating rate of 2.5 °C/min, the infiltrate does not flow into the porous backbone of the final pellets as rapidly and requires vacuuming at a gauge pressure of 68 kPa. This is speculated to be due to graphite completely combusting before LSGM begins to sinter, causing small pores to shrink and close some interconnected pores. At a heating rate of 3.8 °C/min, pellets are prone to cracking during heating. The temperature is held at 1350 °C for 2 h to ensure complete sintering while limiting the time spent at high temperatures. Finally, the sintered pellets are cooled to room temperature at 5 °C/min as faster cooling rates cause cracking.

### Sintered Pellet Microstructure

Cross-sectional scanning electron microscopy (SEM) imaging reveals a successful fabrication of a porous/dense/porous, trilayer structure (Figure 4). Image analysis on the thickness of each layer shows the porous layers shrink from the stacked green tape thickness of 500 to 55 μm (89%) and the dense layer shrinks from a stacked green tape thickness of 200 to 20 μm (90%). Threshold-based image segmentation analysis on the porous layers reveals a porosity of 55%, indicating that the pore diameter shrinks by 10% from the original 65% ceramic percentage for graphite.

**Figure 4:**
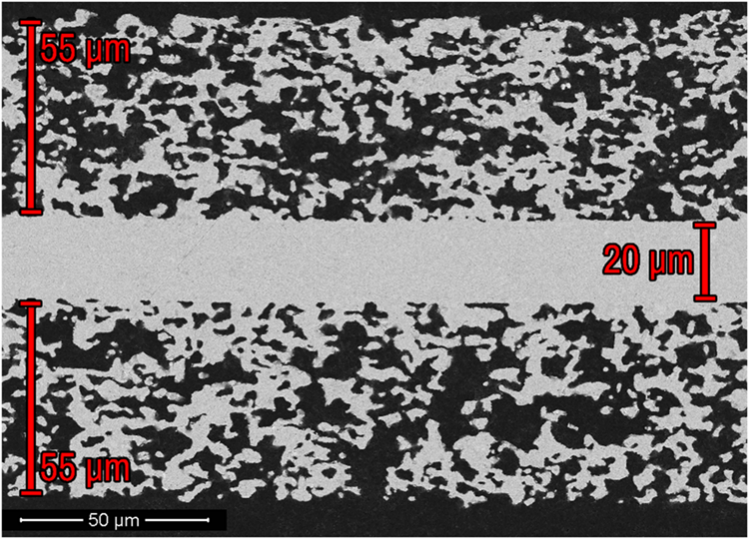
Typical cross-sectional scanning electron microscopy (SEM) micrograph of a porous/dense/porous, trilayer La_0.8_Sr_0.2_Ga_0.8_Mg_0.2_O_3‑δ_ (LSGM) pellet.

### Infiltrate Phase Characterization

High-temperature powder X-ray diffraction (HT-PXRD) of the infiltrated-derived NdBaCoFeO_5+δ_ (NBCF) powder is used to identify the major phases present and assess phase stability across IT-SOFC operating temperatures (Figure 5a). Peak matching of the 23, 600, and 700 °C spectra confirm the major phase is NdBaCoFeO_5+δ_ (*Pmmm*) with BaCO_3_ and tetragonal BaCoO_3‑δ_ (*P*6̅*m*2) being the minor phases. As the temperature reaches 800 °C, the BaCO_3_ phase decomposes, which is expected to yield BaO. Instead, a cubic BaCoO_3‑δ_ phase appears, suggesting that in the presence of Co­(II,III) oxide, BaCO_3_ decomposes to form BaCoO_3–_δ (*Pm̅*3*m*).

**Figure 5:**
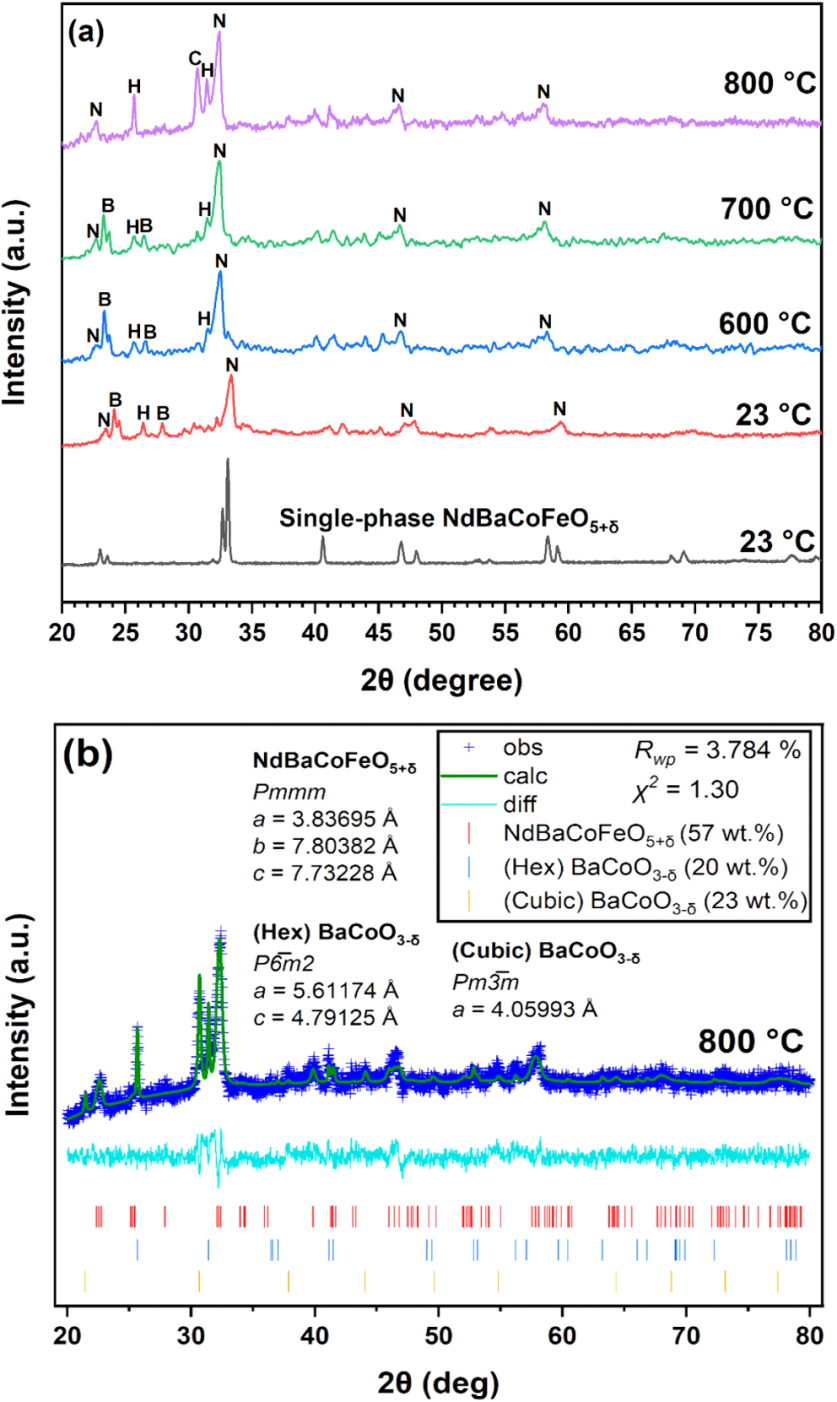
(a) High-temperature powder X-ray diffraction (HT-PXRD) of the NBCF powder prepared by heating a 0.1 M solution of equal stoichiometric amounts of Nd­(III), Ba­(II), Co­(II), and Fe­(III) nitrate at 800 °C for 2 h. The HT-PXRD of the prepared NBCF is measured at room temperature before heating to 600, 700, and 800 °C. N represents the NBCF phase, H is hexagonal BaCoO_3‑δ_, C is cubic BaCoO_3‑δ_, and B is BaCO_3_. (b) Rietveld refinement of a previous HT-PXRD sample prepared at 800 °C in panel (a).

Rietveld refinement of the infiltrate-derived NBCF powder at 800 °C revealed that all detected phases are active for the ORR (Figure 5b). The desired NdBaCoFeO_5+δ_ phase is the major component (57 wt %), while cubic BaCoO_3‑δ_ (23 wt %) and tetragonal BaCoO_3‑δ_ (20 wt %) are present as minor phases^27,28^ Below 800 °C, the low peak intensity and low signal-to-noise ratio prevent reliable Rietveld refinement for determining the precise phase fractions. However, if BaCO_3_ decomposes into the cubic BaCoO_3‑δ_ phase, it can be estimated that approximately 23 wt % of the infiltrated-derived NBCF powder consists of the BaCO_3_ phase at lower temperatures. BaCO_3_ nanoparticles have been shown to decrease the area-specific resistance and be ORR active at ITs.^29^ Additionally, the diffraction peaks of all phases shift to lower 2θ values with increasing temperature, indicating the unit cell expansion and particle growth. Notably, no ORR-active phases exhibit a decrease in the signal intensity at 800 °C, suggesting their stability across the IT-SOFC operating range.

Cross-sectional SEM of the LSGM electrolyte infiltrated with ∼1.58 mg/cm^2^ of NBCF on either side reveals nanoscale particles on the inner pores, with the infiltrated particles reaching the surface of the dense LSGM layer (Figure 6a). To determine whether these particles are NBCF, energy dispersive X-ray spectroscopy spot analysis is used. Locations 1, 2, and 3 on Figure 6a are the cross section of the LSGM backbone, and locations 4, 5, and 6 are the inner pores infiltrated with NBCF. Figure 6b shows that locations 1, 2, and 3 feature only the elements present in LSGM. However, the atomic concentration slightly overestimates the concentration of La and Ga from the original La_0.8_Sr_0.2_Ga_0.8_Mg_0.2_O_3‑δ_ stoichiometry. As La and Ga emit higher energy X-rays, they may be less affected by surface roughness when compared to Sr and Ga leading to a more intense La and Ga signal.^30^ Spot analysis inside the LSGM pores shows the addition of Nd, Ba, Co, and Fe signals, indicating that NBCF nanoparticles are infiltrating deep into the porous LSGM backbone (Figure 6c). Ba appears to have a higher concentration than Nd, Co, and Fe despite infiltrating equal stoichiometric quantities. This is likely caused by the Lα emission of La and Ba being separated by ∼0.18 keV, leading to overlapping signals and the overestimation of Ba and underestimation of La.

**Figure 6:**
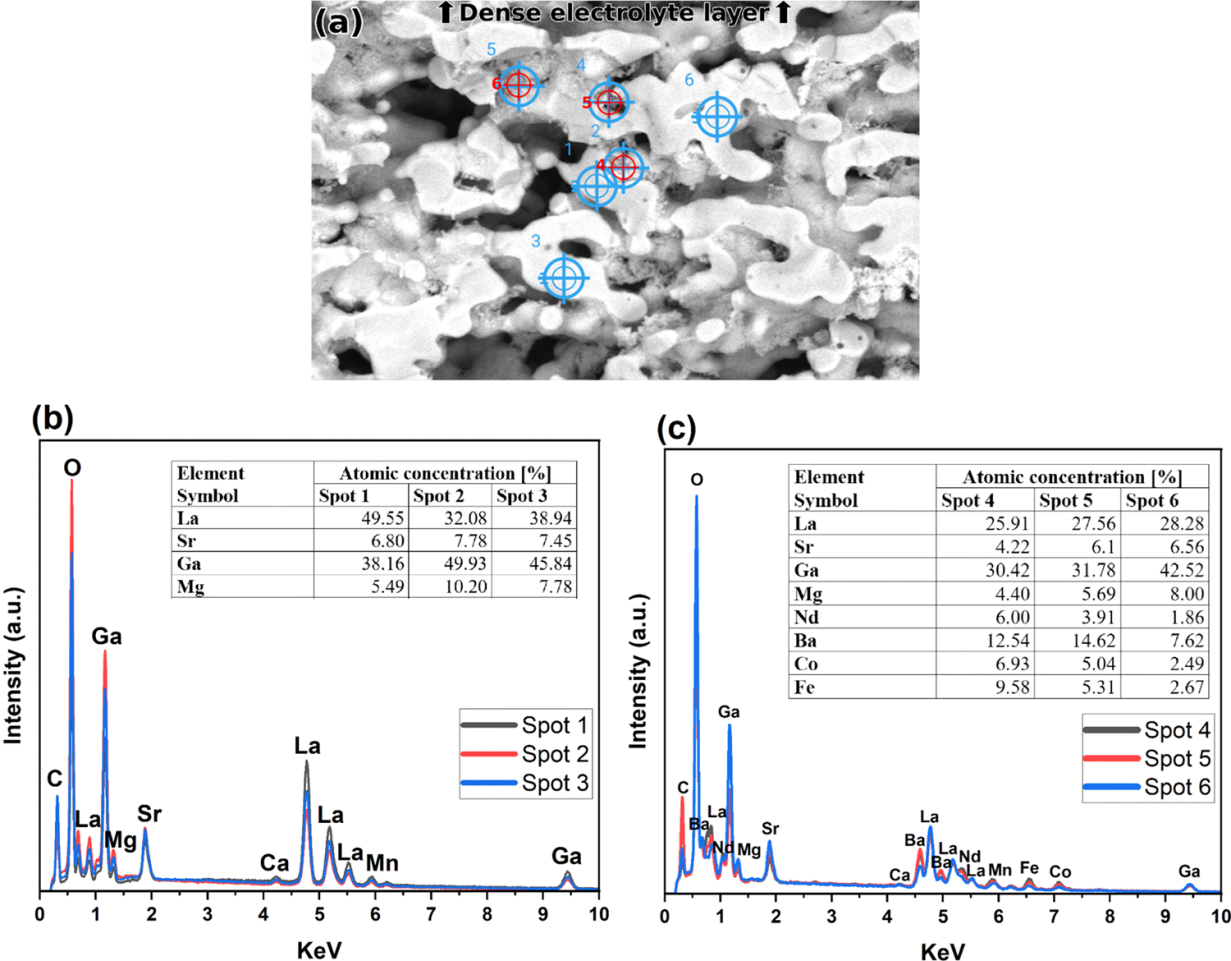
(a) Cross-sectional SEM backscatter micrograph (15 kV acceleration voltage, 13,000× magnification) of La_0.8_Sr_0.2_Ga_0.8_Mg_0.2_O_3‑δ_ (LSGM) infiltrated with ∼1.58 mg/cm^2^ of NBCF on either side. Energy dispersive X-ray spectroscopy spot analysis of Figure 6a for (b) spots 1, 2, and 3 of the cross section of the LSGM backbone and (c) spots 4, 5, and 6 of the inner pores infiltrated with NBCF. The C signal from the carbon tape, the O signal from the perovskite, and the Ca and Mn contamination signals are removed from the atomic concentration.

### Symmetric Cell Electrochemical Characterization

Electrochemical impedance spectroscopy (EIS) is used to determine the catalytic activity of LSGM symmetric cells infiltrated with various masses per unit area of NBCF (Figure 7a–c). The area-specific resistance (ASR) is used to quantify the catalytic of symmetric NBCF cells using the formula

6 $$\mathrm{ASR}=\frac{R_{p}\times A}{2}$$

where *R*_p_ is the polarization resistance equal to the low-frequency *Z*′(Ω) intercept of an ohmic resistance subtracted Nyquist plot and *A* is area (cm^2^). The polarization resistance is halved to account for the resistance of NBCF in a full asymmetric cell. At 1.05 mg/cm^2^ of loaded NBCF, ASR values of 0.345, 0.144, 0.088, 0.059, and 0.042 Ω cm^2^ are obtained at 600, 650, 700, 750, and 800 °C respectively. Increasing the loaded NBCF to 1.58 mg/cm^2^ lowers the ASR values to 0.174, 0.078, 0.041, 0.040, and 0.025 Ω cm^2^ at 600, 650, 700, 750, and 800 °C respectively. Further increasing the loaded NBCF to 2.11 mg/cm^2^ results in the largest ASR values of 0.40, 0.265, 0.145, 0.085, and 0.063 Ω cm^2^ at 600, 650, 700, 750, and 800 °C respectively.

**Figure 7:**
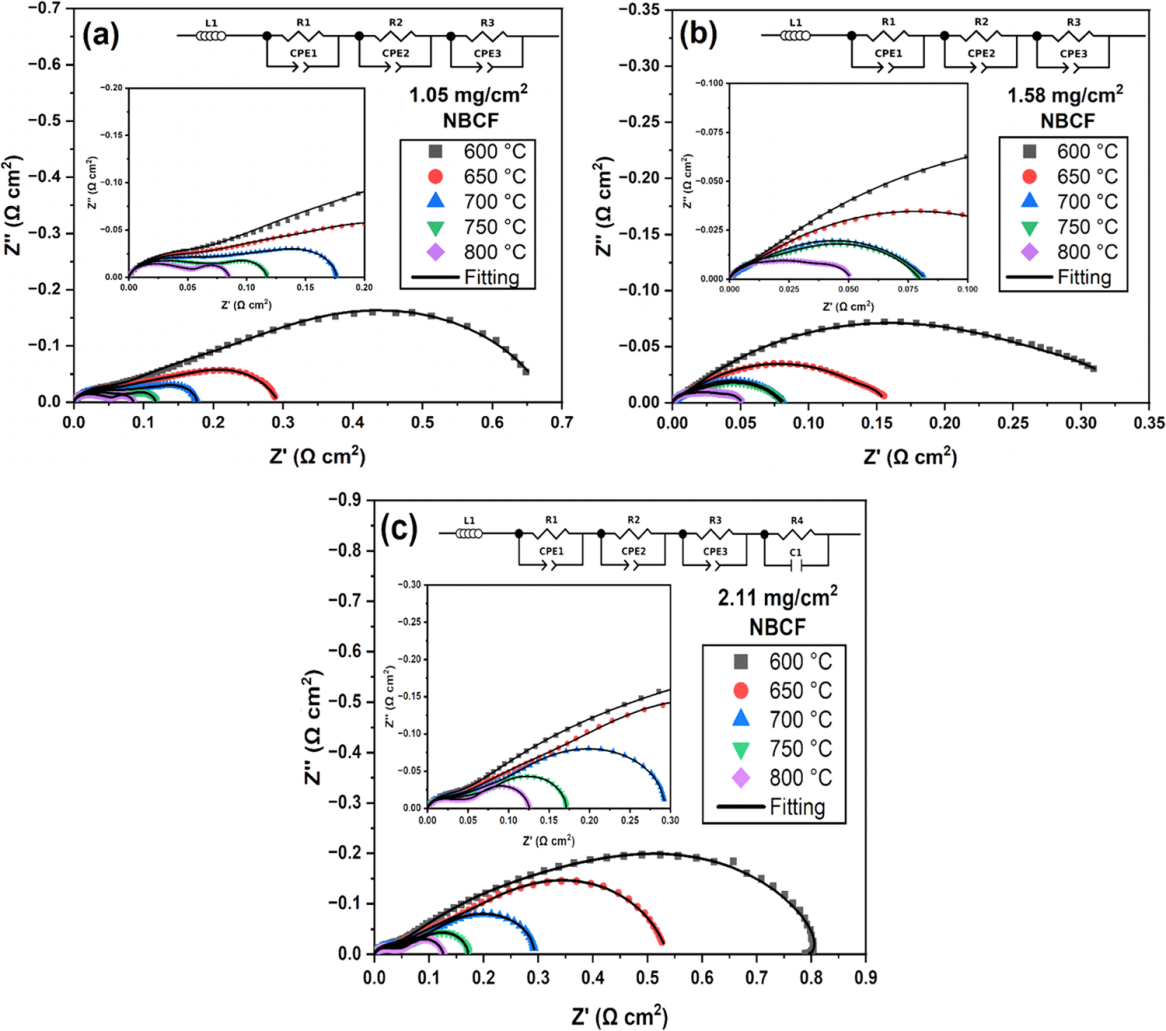
An area-corrected (0.95 cm^2^) Nyquist plot of electrochemical impedance spectra (EIS), with ohmic resistance removed, measured in an ambient air at 600–800 °C with an amplitude of 10 mV over a frequency range of 10^6^–10^–2^ Hz for symmetric cells infiltrated with (a) 1.05 mg/cm^2^, (b) 1.58 mg/cm^2^, and (c) 2.11 mg/cm^2^ of NBCF particles loaded into the porous backbone of a trilayer (porous/dense/porous) LSGM pellet (Figure 4). Fitting is from an equivalent circuit model generated from a distribution of relaxation times and least-squares fitting.^31^

A distribution of relaxation times (DRT) is generated from the EIS results to deconvolute the spectrum and separate the total ASR into contributions from different frequency-dependent processes: low-frequency (LF) diffusion resistance (<10 Hz), mid-frequency (MF) dissociative adsorption resistance (10–10^3^ Hz), and high frequency (HF) charge transfer resistance (>10^3^ Hz) (Figure 8a–c).^32–35^ At an NBCF loading of 1.05 mg/cm^2^, the DRT shows a sharp peak centering at 1 Hz at 600 °C, which shifts to 5 Hz at 800 °C; a broad peak centering at 11 Hz at 600 °C, which shifts to 400 Hz at 800 °C; and a sharp peak centering at 15 kHz at 600 °C, which shifts to 5.5 kHz at 800 °C. At an NBCF loading of 1.58 mg/cm^2^, the DRT shows: a broad peak centering at 0.8 Hz at 600 and 650 °C, which reduces to a negligible area at 700 °C and above; a sharp peak centering 11 Hz at 600 °C, which shifts to 50 Hz at 750 °C, then splits into two broad peaks centering at 12 and 180 Hz at 800 °C; and a peak centering at 5 kHz at 600 °C, which shifts to 2 kHz at 800 °C and sharpens. At an NBCF loading of 2.11 mg/cm^2^, the DRT shows: a sharp peak centering at 1.5 Hz at 600 °C, which shifts to 9 Hz at 800 °C; a broad peak centering at 25 Hz at 600 °C, which shifts to 400 Hz at 800 °C; and a peak centering at 25 kHz at 600 °C, which shifts to 12 kHz at 800 °C. The shifts to higher frequency with increasing temperature for the LF diffusion and MF dissociative adsorption processes indicate faster kinetics for those steps of the ORR. In contrast, the shifts to lower frequency with increasing temperature for the HF charge transfer processes suggest slower kinetics. This decrease in HF charge transfer frequency aligns with previous findings, which demonstrate an inverse proportionality between conductivity and temperature for similar materials above 500 °C.^18^

**Figure 8:**
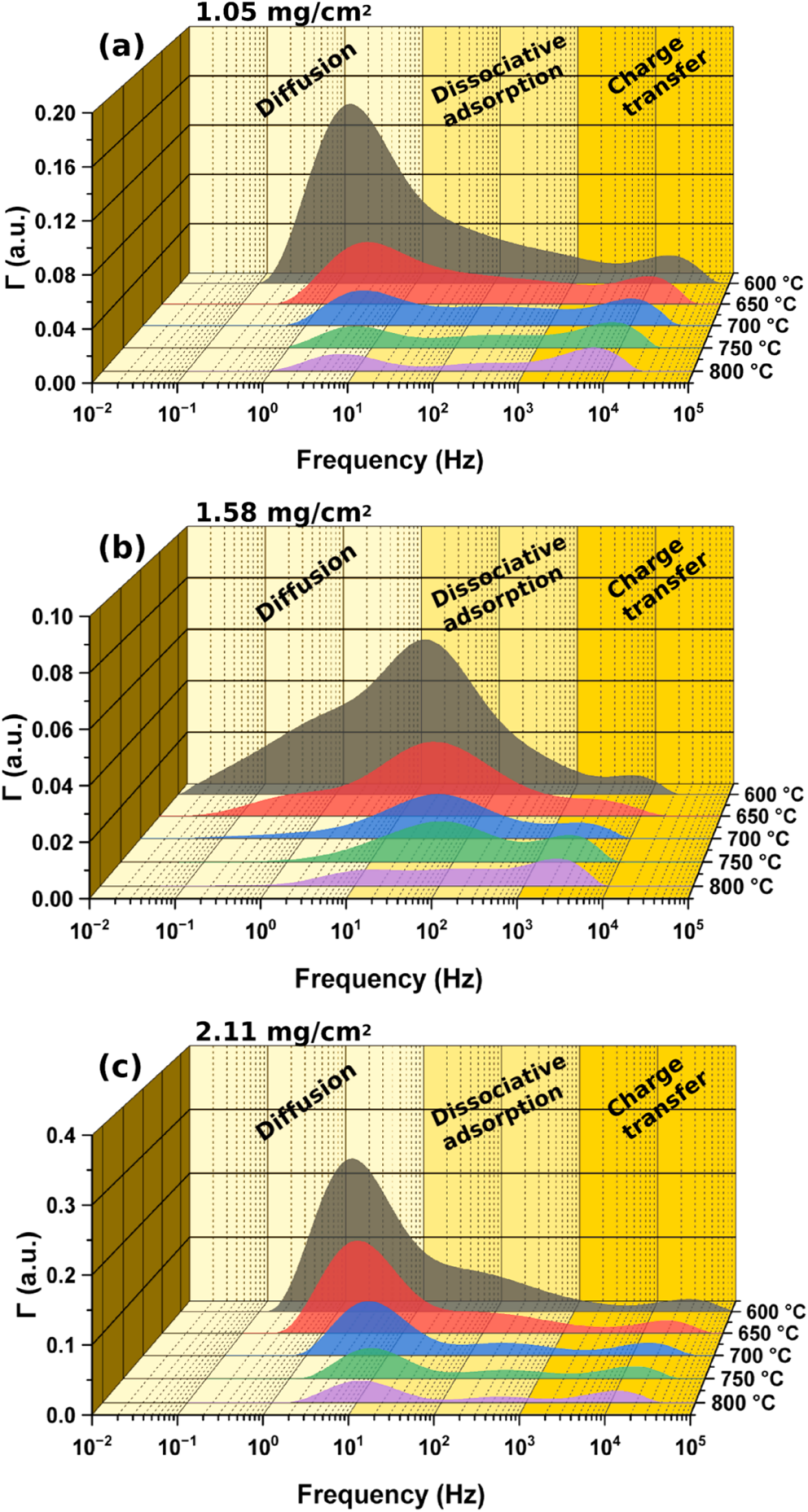
Distribution of relaxation times (DRT) frequency domain plot of (a) 1.05 mg/cm^2^, (b) 1.58 mg/cm^2^, and (c) 2.11 mg/cm^2^ of NBCF particles loaded into the porous backbone of a trilayer (porous/dense/porous) LSGM. DRT is generated using DRT tools with a Gaussian radial basis function for discretization, a first derivative Γ­(τ) penalty term to stabilize DRT oscillations, a regularization parameter of 8.13 × 10^–3^, and a full-width-half-maximum coefficient of 0.5.^25,26^

Using the area of the deconvoluted DRT peaks and subsequent equivalent circuit (EC) modeling, the resistance of each process can be determined (Table 2). The lowest overall resistance occurs at an NBCF loading of 1.58 mg/cm^2^, which is attributed to the reduction in both the HF charge transfer resistance (*R*_1_) and the LF diffusion resistance (*R*_3_) compared to loadings of 1.05 and 2.11 mg/cm^2^. At the lower NBCF loading (1.05 mg/cm^2^), there are too few NBCF particles on the LSGM backbone surface, leading to insufficient connectivity for electron conduction and an increase in the level of *R*_1_. Additionally, *R*_3_ also increases due to the limited availability of NBCF particles, resulting in surface saturation with adsorbed oxygen, which inhibits the dissociated oxygen from diffusing to the TPB. At a higher NBCF loading (2.11 mg/cm^2^), the NBCF particles likely agglomerate, creating longer diffusion pathways from ORR-active sites to the TPB, thereby increasing *R*_3_. Additionally, the formation of thicker NBCF layers reduces the conductivity, leading to an increase in *R*_1_. The MF dissociative adsorption resistance (*R*_2_) increases with increasing NBCF loading, indicating a hindrance of oxygen vacancy availability, which affects both adsorption and dissociation processes. A LF capacitance loop (*R*_4_|*C*_1_) is introduced in the EC model for the 2.11 mg/cm^2^ NBCF loading at 600, 650, and 700 °C. This phenomenon occurs at higher NBCF loadings due to the increased number of oxygen adsorption sites. Previously, studies have reported that such behavior in nanoparticle electrodes is linked to oxygen adsorption processes, which result in negative resistance and a negative capacitance impedance response.^36–38^ However, at 750 and 800 °C, the capacitance loop is not visible within the measured frequency range.

**Table 2:** Equivalent Circuit Parameters from Figure 7a–c Solved for Using Sequential Least-Squares Fitting

	infiltrate mass (mg/cm^2^)	*L*_1_ (nH/cm^2^)	*R*_1_ (Ω cm^2^)	CPET_1_ (F/cm^2^)	CPEP_1_	*R*_2_ (Ω cm^2^)	CPET_2_ (F/cm^2^)	CPEP_2_	*R*_3_ (Ω cm^2^)	CPET_3_ (F/cm^2^)	CPEP_3_
600 °C	1.05	5.28	0.062	0.015	0.63	0.257	0.245	0.53	0.371	0.457	0.79
	1.58	10.88	0.013	0.178	0.48	0.296	0.298	0.56	0.039	16.99	0.77
	2.11	9.33	0.078	0.041	0.45	0.418	0.138	0.56	0.372	0.289	0.78
650 °C	1.05	13.06	0.072	0.028	0.57	0.121	0.297	0.55	0.113	0.684	0.81
	1.58	13.50	0.025	0.868	0.30	0.126	0.392	0.57	0.026	16.89	0.44
	2.11	14.44	0.095	0.085	0.38	0.225	0.116	0.66	0.284	0.301	0.89
700 °C	1.05	13.82	0.063	0.054	0.54	0.076	0.600	0.47	0.050	0.867	0.88
	1.58	12.46	0.012	0.108	0.62	0.072	0.421	0.63			
	2.11	11.82	0.045	0.0108	0.63	0.097	0.286	0.54	0.186	0.290	0.83
750 °C	1.05	18.38	0.080	0.058	0.51	0.027	0.790	0.64	0.026	1.258	0.95
	1.58	18.52	0.020	0.047	0.67	0.064	0.352	0.62			
	2.11	15.17	0.059	0.056	0.54	0.059	0.342	0.59	0.073	0.227	0.97
800 °C	1.05	18.97	0.059	0.033	0.58	0.011	0.511	0.72	0.027	1.321	0.89
	1.58	17.69	0.013	0.018	0.77	0.029	0.259	0.63	0.013	2.226	0.80
	2.11	16.84	0.052	0.019	0.57	0.031	0.442	0.58	0.059	0.334	0.93

### Full Cell Electrochemical Characterization

The total resistance of a trilayer LSGM electrolyte infiltrated with ∼1.58 mg/cm^2^ NBCF at the cathode and ∼1.24 mg/cm^2^ Ni-GDC at the anode under SOFC operating conditions is determined using EIS (Figure 9a). The HF *Z*′ intercept corresponds to the ohmic resistance (*R*_o_) from LSGM. With a dense LSGM electrolyte layer thickness of 20 μm and a total porous LSGM layer thickness of 110 μm, the cell exhibits *R*_o_ values of 0.354, 0.230, 0.137, 0.111, and 0.098 Ω cm^2^ at 600, 650, 700, 750, and 800 °C, respectively. The LF *Z*′ intercept corresponds to the polarization resistance (*R*_p_) from the LF gas diffusion resistance, MF electrochemical redox resistance, and HF charge transfer resistance at both the cathode and anode. With ∼1.58 mg/cm^2^ NBCF at the cathode and ∼1.24 mg/cm^2^ Ni-GDC at the anode, the cell exhibits *R*_p_ values of 3.74, 1.066, 0.449, 0.334, and 0.289 Ω cm^2^ at 600, 650, 700, 750, and 800 °C, respectively.

**Figure 9:**
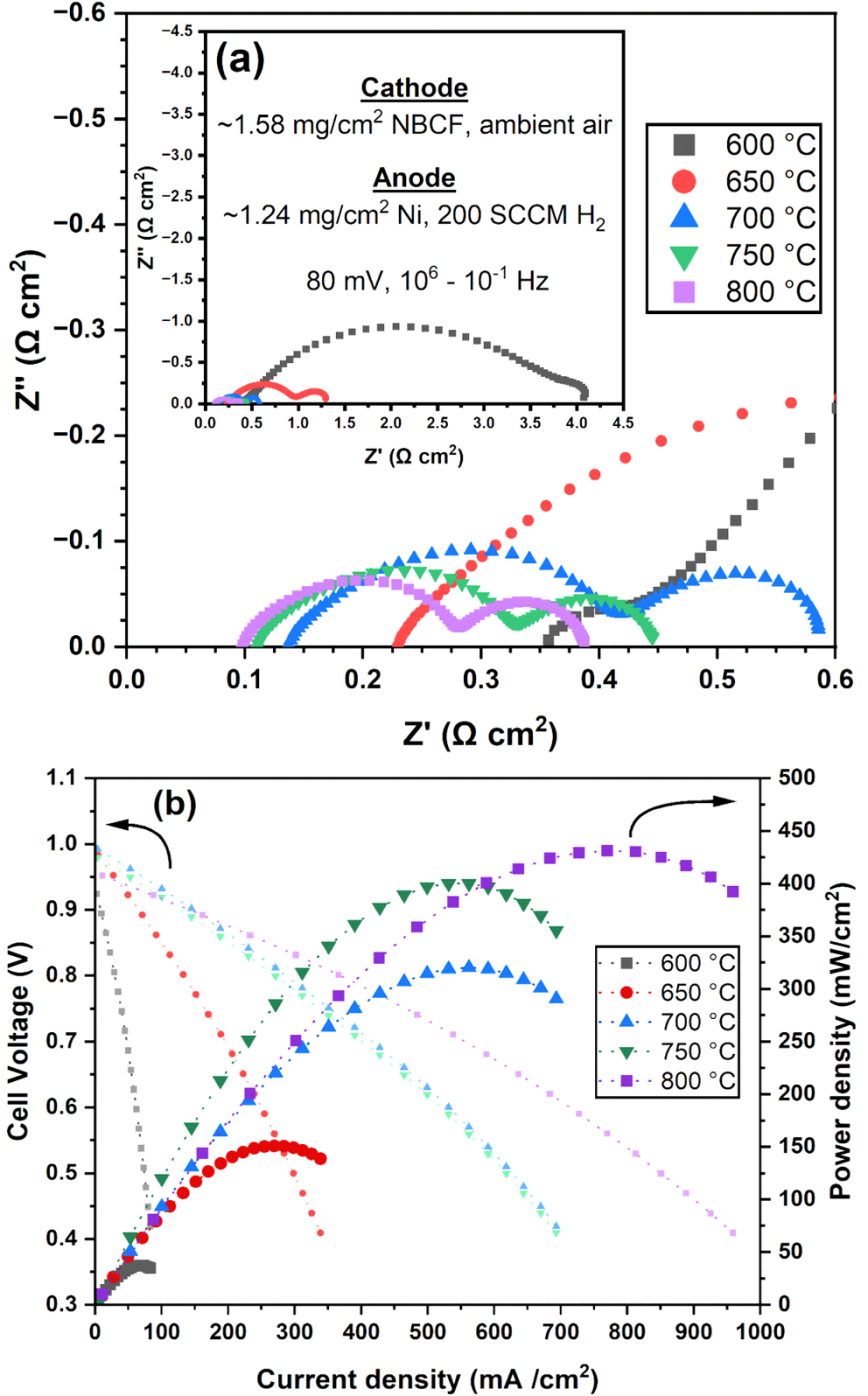
(a) Area-corrected (0.95 cm^2^) Nyquist plot of the EIS data for a full cell with trilayer LSGM electrolyte infiltrated with ∼1.58 mg/cm^2^ NBCF cathode and ∼1.24 mg/cm^2^ Ni-GDC anode into the porous LSGM backbone. Measurements are performed in ambient air at the cathode with H_2_ supplied at 200 SCCM to the anode at 600–800 °C, using a 20 mV AC amplitude over a frequency range of 10^6^–10^–1^ Hz. (b) Current density–voltage (IV) plot of the same cell, tested in the ambient air at the cathode with H_2_ supplied at 200 SCCM at the anode at 600–800 °C. IV measurements are conducted from open-circuit potential (OCV) to 0.4 V, with a step weight of 30 mV and a scan rate of 5 mV/s. The lighter-shaded plot represents voltage as a function of current density, while the darker-shaded plot corresponds to power density as a function of current density.

Current density–voltage (IV) measurement of the trilayer LSGM electrolyte is performed to determine the peak powder density (PPD) and benchmark the overall performance of the full cell (Figure 9b). The cell achieves PPD values of 37, 151, 320, 400, and 431 mW/cm^2^ and reaches at 600, 650, 700, 750, and 800 °C, respectively. For both the symmetric NBCF cells and NBCF/Ni-GDC full cells, a significant drop in resistance occurs between 600 and 700 °C, resulting in a 283 mW/cm^2^ increase in PPD, followed by a more gradual decrease between 700 and 800 °C, resulting in a 111 mW/cm^2^ increase. Compared to literature values at 750 °C, the trilayer LSGM electrolyte infiltrated with ∼1.58 mg/cm^2^ NBCF at the cathode and ∼1.24 mg/cm^2^ Ni-GDC at the anode exhibit lower or comparable *R*_o_ and *R*_p_ reducing power losses due to internal resistance (Table 3). However, despite these low resistances, the PPD at 800 °C remains lower than expected. Since the gas adsorption/desorption and charge transfer resistances for NBCF symmetric cells at 1.58 mg/cm^2^ are low, and the internal resistance of the full cell is also low, the activation and ohmic overpotentials are accordingly low.^3^ This indicates that the concentration overpotential is relatively high, which limits the PPD. To address this, additional NBCF mass loading may be required to achieve a higher PPD despite the increased *R*_p_ observed in symmetric cells with higher NBCF content. Alternatively, further Ni-GDC mass loading may be necessary to enhance performance.

**Table 3:** Comparison of the Full Cell Electrochemical Performance for SOFCs with an Infiltrated Anode and Cathode

cell configuration (cathode/electrolyte/anode)	electrolyte thickness (μm)	*R*_o_ (Ω cm^2^)	*R*_p_ (Ω cm^2^)	PPD (mW/cm^2^)	refs
NBCF/LSGM/Ni-GDC (LSGM ring support)	55 (porous + ring), 20 (dense), 55 (porous + ring)	0.11 (750 °C)	0.33 (750 °C)	400 (750 °C)	this work
LSF/YSZ/LSV (YSZ support)	112 (support), 48 (porous), 55 (dense), 48 (porous), 112 (support)	∼0.45 (800 °C)	∼0.52 (800 °C)	245 (800 °C)	^39^
LSM/YSZ/Ni (YSZ support with printed LSM)	50 (porous LSM), 150 (dense), 25 (porous)	∼0.98 (800 °C)	∼ 1.12 (800 °C)	∼ 400 (800 °C)	^40^
LSCF/YSZ/Ni (LSCF-YSZ support)	700 (support), 20 (porous), 15 (dense), 20 (porous)	0.46 (750 °C)	0.57 (750 °C)	378 (750 °C)	^41^
LSCF/YSZ/Ni (Ni-YSZ support)	30 (porous), 30 (dense), 30 (support), 30 (porous)	0.35 (750 °C)	1.45 (750 °C)	∼ 350 (750 °C)	^42^
LSCF/YSZ/Pd-SDC (YSZ support)	50 (porous), 40 (dense), 300 (porous support)	0.19 (750 °C)	0.19 (750 °C)	733 (750 °C)	^43^

## Conclusions

This work demonstrates the fabrication of a trilayer (porous/dense/porous) La_0.8_Sr_0.2_Ga_0.8_Mg_0.2_O_3−δ_ electrolyte using an optimized tape casting method. The resulting structure consists of porous layers with a thickness of 55 μm and 55% porosity, a dense thickness of 20, and 55 μm thick LSGM support rings. The binder/plasticizer ratio is optimized to produce strong, defect-free green tapes, while thermogravimetric analysis (TGA) is used to refine the sintering profile, enabling a well-structured LSGM pellet to be achieved through a single-step sintering process. The porous LSGM backbone is infiltrated with a NdBaCoFeO_5+δ_ (NBCF) cathode and Ni–Gd-doped-Ce (Ni-GDC) anode for symmetric NBCF and full cell testing. Rietveld refinement of powder X-ray diffraction (PXRD) at 800 °C reveals that the NBCF powder consists of 57 wt % NBCF, 23 wt % cubic BaCoO_3−δ_, and 20 wt % hexagonal BaCoO_3−δ_, with BaCO_3_ phases appearing at ≤750 °C. Electrochemical impedance spectroscopy (EIS), distribution of relaxation times (DRT), and equivalent circuit (EC) modeling for symmetrical NBCF cells reveals that the lowest overall resistance occurs at an NBCF loading of 1.58 mg/cm^2^, minimizing both charge transfer and diffusion resistances and reducing the area-specific-resistance (ASR) to 0.025 Ω cm^2^ at 800 °C. However, increasing the NBCF loading to 2.11 mg/cm^2^ leads to particle agglomeration, which increases diffusion resistance and reduces performance. Full cell testing with ∼1.58 mg/cm^2^ NBCF in ambient air and ∼1.24 mg/cm^2^ Ni supplied with 200 SCCM of H_2_ produces a peak powder density (PPD) of 400 mW/cm^2^ at 750 °C, with an ohmic resistance of 0.11 Ω cm^2^ and polarization resistance of 0.33 Ω cm^2^. Despite these resistances being lower or comparable to values reported in the literature, the power output does not reach expectations at 800 °C due to high concentration overpotential relative to the activation and ohmic overpotentials. This suggests that additional infiltrate loading at either the anode or cathode may be necessary to enhance performance. Overall, this work highlights the effectiveness of tape casting for producing thin, durable trilayer electrolyte structures with the potential for cost-effective electrode infiltration of nanoparticles into the porous electrolyte backbone.
